# A tribute to Cecilio Romaña: Romaña’s sign in Chagas disease

**DOI:** 10.1371/journal.pntd.0008836

**Published:** 2020-11-12

**Authors:** Nathan Beucler, Faustino Torrico, David Hibbert

**Affiliations:** 1 Neurosurgery Department, Sainte-Anne Military Teaching Hospital, Toulon, France; 2 Ecole du Val-de-Grâce, Paris, France; 3 Faculty of Medicine, Universidad Mayor de San Simon, Cochabamba, Bolivia; 4 Department of Internal Medicine, UC San Diego Medical Center, San Diego, California, United States of America; George Washington University School of Medicine and Health Sciences, UNITED STATES

## Introduction

**Fig 1:**
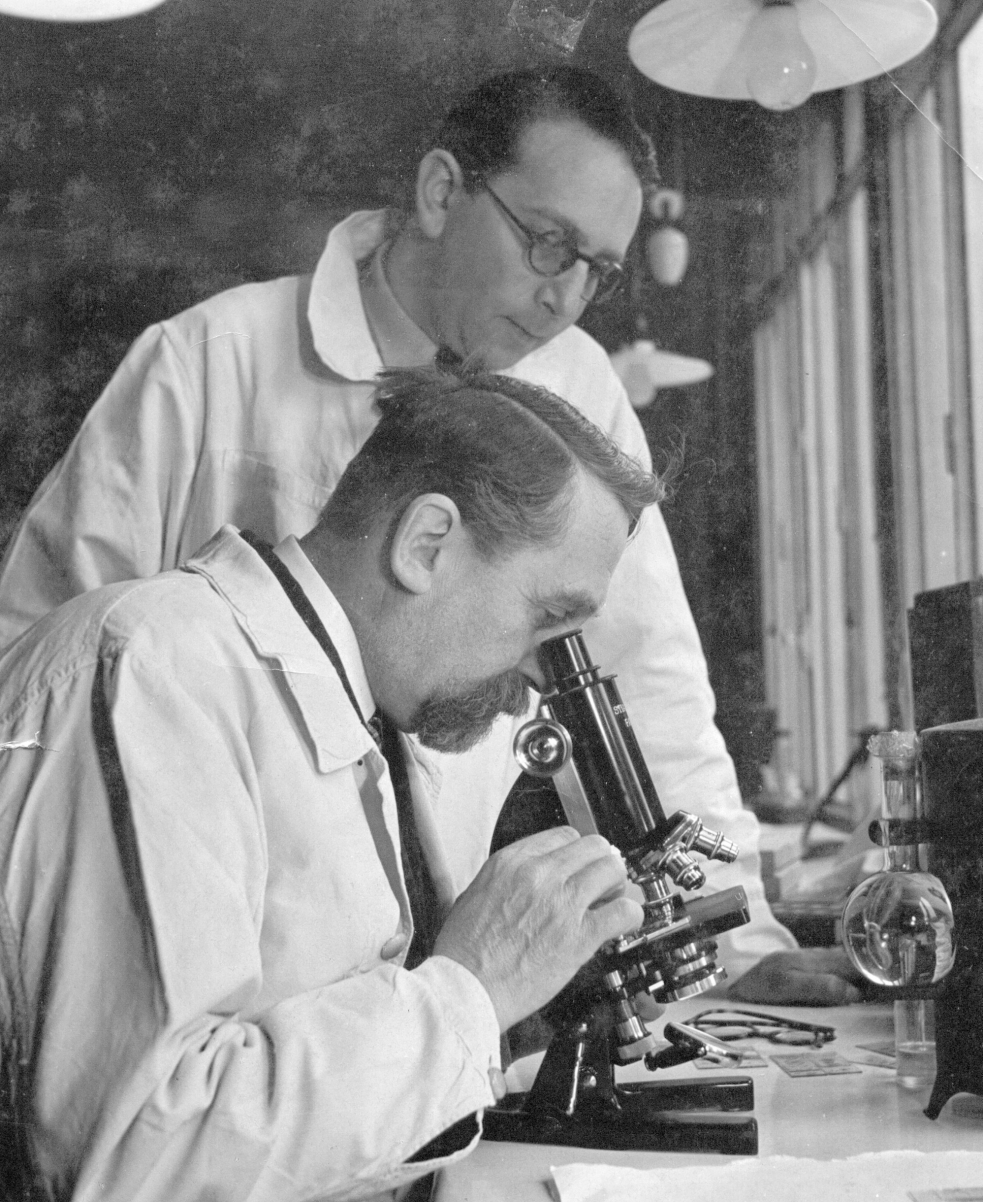
Cecilio Romaña working with Emile Roubaud.

**Fig 2:**
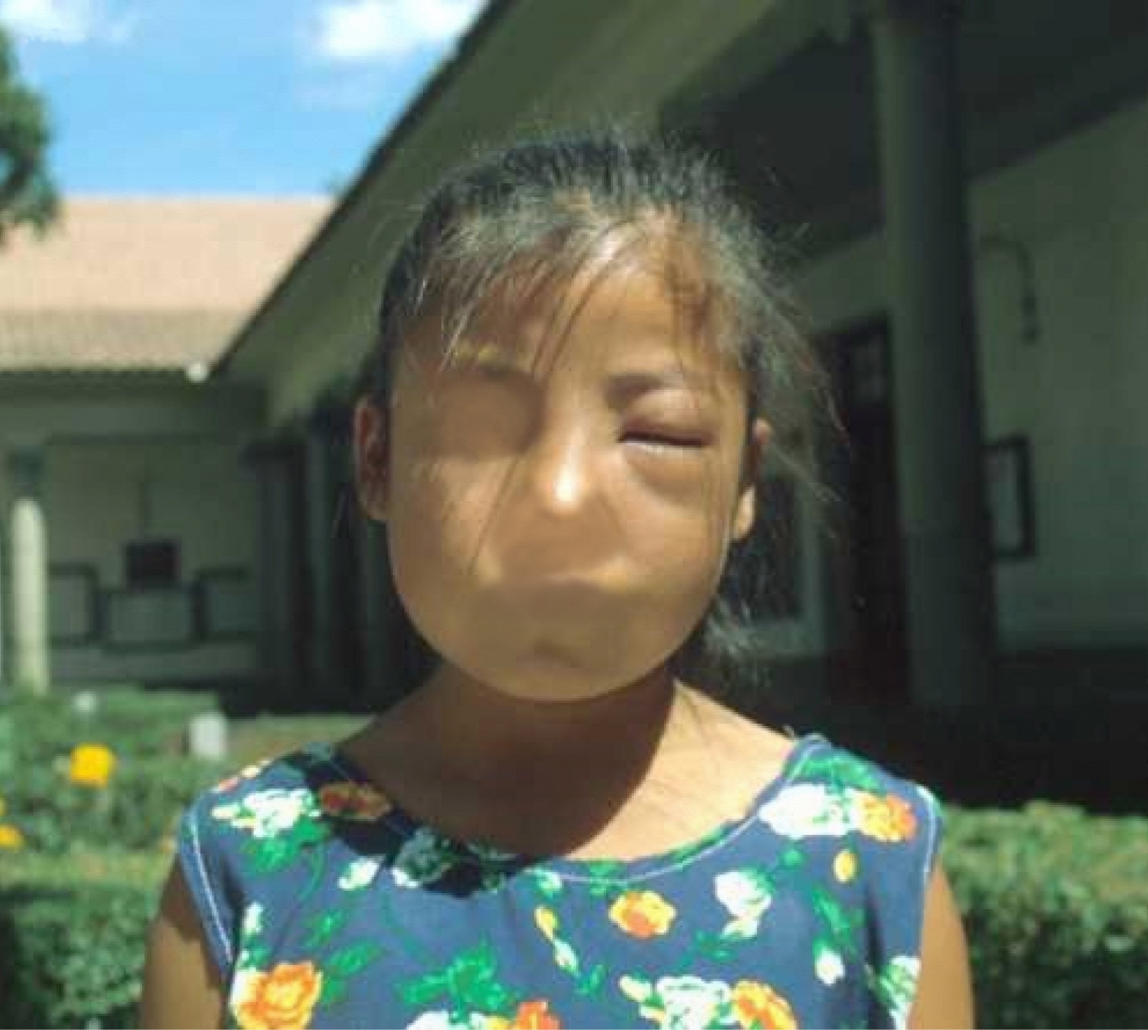
Girl with Romaña’s sign taken in the courtyard of the Faculty of Medicine, Universidad Mayor de San Simón, Bolivia around 1980.

Chagas disease refers to the infection by the parasite *Trypanosoma cruzi*, transmitted by the bite of species of Triatoma, also known as the kissing bug. The disease plagued South America at a pandemic scale during the early 20th century [1]. At that time, a few researchers devoted themselves to this infection, like Carlos Chagas, Emmanuel Dias, Emile Roubaud, or even Cecilio Romaña (Fig 1). The latter described the transmission pathway through the conjunctiva that leads to periorbital painless swelling, which is known as the Romaña’s sign and indicates acute Chagas disease (Fig 2) [2].

## Testimony

During a consultation in the middle of the night in the emergency department of Sainte-Anne Military Teaching Hospital in France, we incidentally met Louis Alexandre Romaña, son of Cecilio Romaña. By personal curiosity, we asked him about his father.

**Fig 3:**
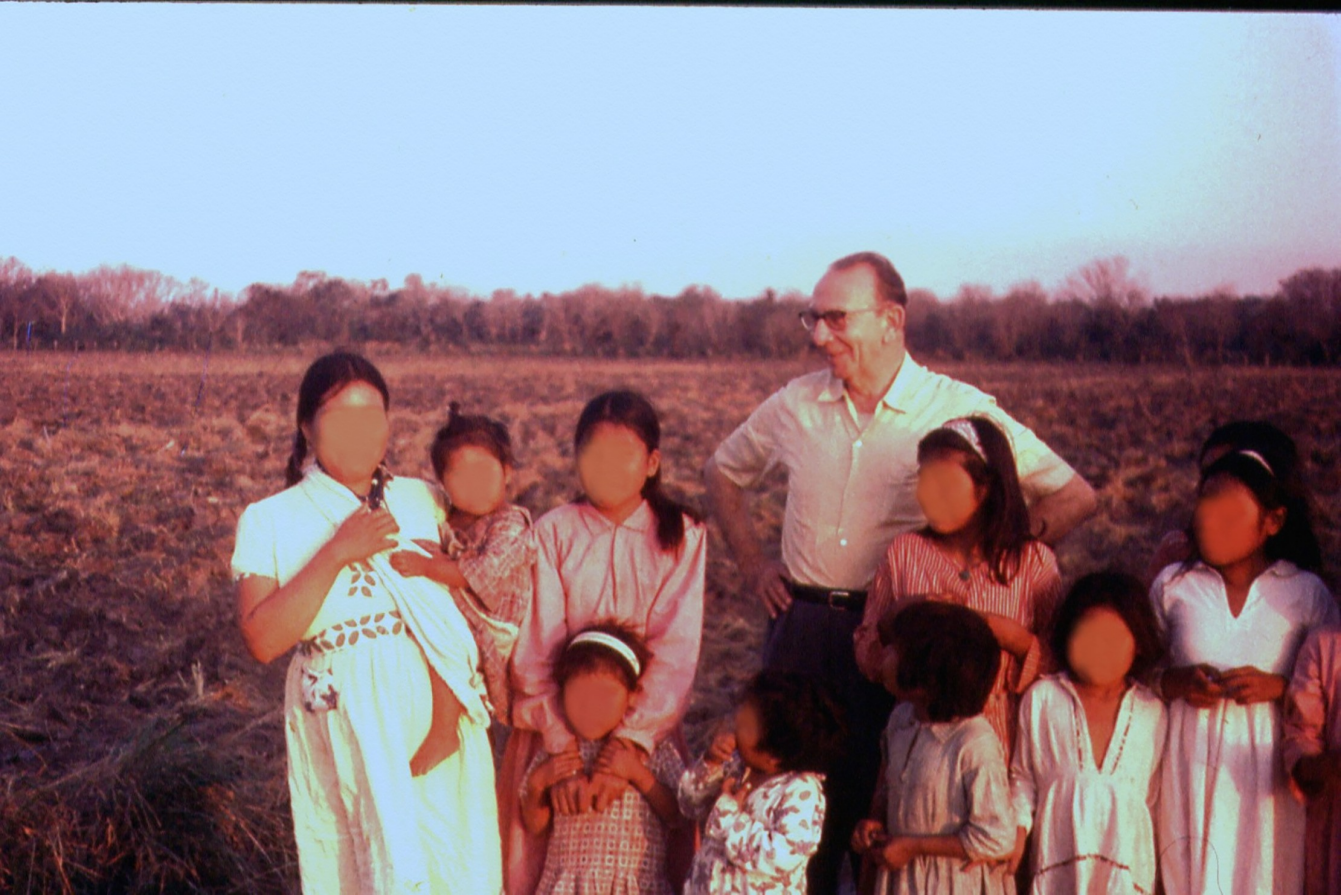
Cecilio Romaña in the province of Chaco, Argentina.

“Dr. Cecilio Romaña is known to the world for Romaña’s sign, but to me, he was my father. Beyond his interests in medicine, he was a sculptor, a writer, and a researcher in northern Argentina. At that time, there was an extensive indigenous population working in the logging industry who were falling severely ill to Chagas disease on an epidemic scale (Fig 3). He began studying the disease and met with Evandro Chagas, the son of Carlos Chagas, in Brazil. Through his research he discovered the periorbital swelling syndrome (1935), which is one of the initial signs of *Trypanosoma cruzi* invading the bloodstream.

During those times, people slept in thatched cottages which provided an environment that was particularly hospitable for the Reduviid bug, also known as the kissing bug, for its characteristic blood-sucking bite on the face. At night this insect would climb down from the roof, bite the inhabitant’s face and defecate the *T*. *cruzi* parasite. The bite is irritating to the skin which would cause people to scratch and rub the stool into their eye allowing the trypanosome parasite to penetrate through the conjunctiva which causes inflammation. The Brazilians decided that it would be called Romaña’s sign in honor of his discovery.”

                    In memory of Dr. Cecilio Romaña, 1899–1997 (S1 Text)

                              Louis Alexandre Romaña, son of Cecilio Romaña

                                                  July 31, 2019

## Discussion

Because of vector control, acute Chagas disease with Romaña’s sign has become a rarity, even in South America [3]. These pictures remind us of 50 years of tropical medicine, from Romaña’s discovery in the 1930s as recounted by his son to a girl with acute Chagas disease in Bolivia in the 1980s.

## Permission to reuse material

Louis-Alexandre Romaña has granted permission for the reuse of the photographs that he owns and that he has provided (Figs 1 and 3) and for his letter. Written permission is included as Supporting information.

Dr. Faustino TORRICO has granted permission for the reuse of the photograph that he owns and that he has provided (Fig 2).

## Supporting information

S1 Text Original text in French. (DOCX)

S1 Authorization Authorization to publish testimony. (JPG)
