# Supplementary material for: A tribute to Cecilio Romaña: Romaña’s sign in Chagas disease
Source: PLoS Negl Trop Dis. 2020 Nov 12;14(11):e0008836. doi: 10.1371/journal.pntd.0008836 (PMC7660534; doi:10.1371/journal.pntd.0008836)
Supplement: S1 Text — (DOCX) [file pntd.0008836.s001.docx]

**Original text in French**

« Cecilio Romaña, qui était mon père, qui était donc médecin tropicaliste, décédé il y a déjà pas mal de temps. C’était un grand humaniste, en plus d’être médecin : il était sculpteur, écrivain, et puis il a fait de la recherche à l’époque où il se trouvait au nord de l’Argentine, comme médecin dans une entreprise anglaise qui travaillait le tanin, où il y avait une quantité phénoménale de bûcherons, beaucoup de bûcherons indiens qui étaient là-bas. Et peu-à-peu il s’est rendu compte que ce n’était pas quelques chose de commun la maladie de Chagas, que c’était quand même une pandémie. Il a commencé à faire des études sur la maladie, il a commencé à discuter avec Evandro Chagas, qui était le fils de Carlos Chagas au Brésil. Et puis, peu-à-peu, il a découvert le syndrome palpébral (1935), comme vous dites, qui est à l’origine en fait de beaucoup de la pénétration du parasite, les *Trypanosoma cruzi*, dans la circulation sanguine. En général, les gens dorment dans des chaumières. La vinchuca, qui est un insecte hématophage, pique le soir, descend, et déjecte. En même temps qu’il fait sa déjection, il déjecte aussi le trypanosome ; les gens se grattent, et quand ils se réveillent ils se frottent les yeux, et le trypanosome pénètre par la muqueuse oculaire.

C’est les brésiliens qui ont décidé que ça s’appellerait le syndrome de Romaña.

A la mémoire de Cecilio Romaña, 1899–1997 Barcelone »

Louis Alexandre Romaña

31 Juillet 2019
