# Supplementary figures and images for: A tribute to Cecilio Romaña: Romaña’s sign in Chagas disease
Source: PLoS Negl Trop Dis. 2020 Nov 12;14(11):e0008836. doi: 10.1371/journal.pntd.0008836 (PMC7660534; doi:10.1371/journal.pntd.0008836)

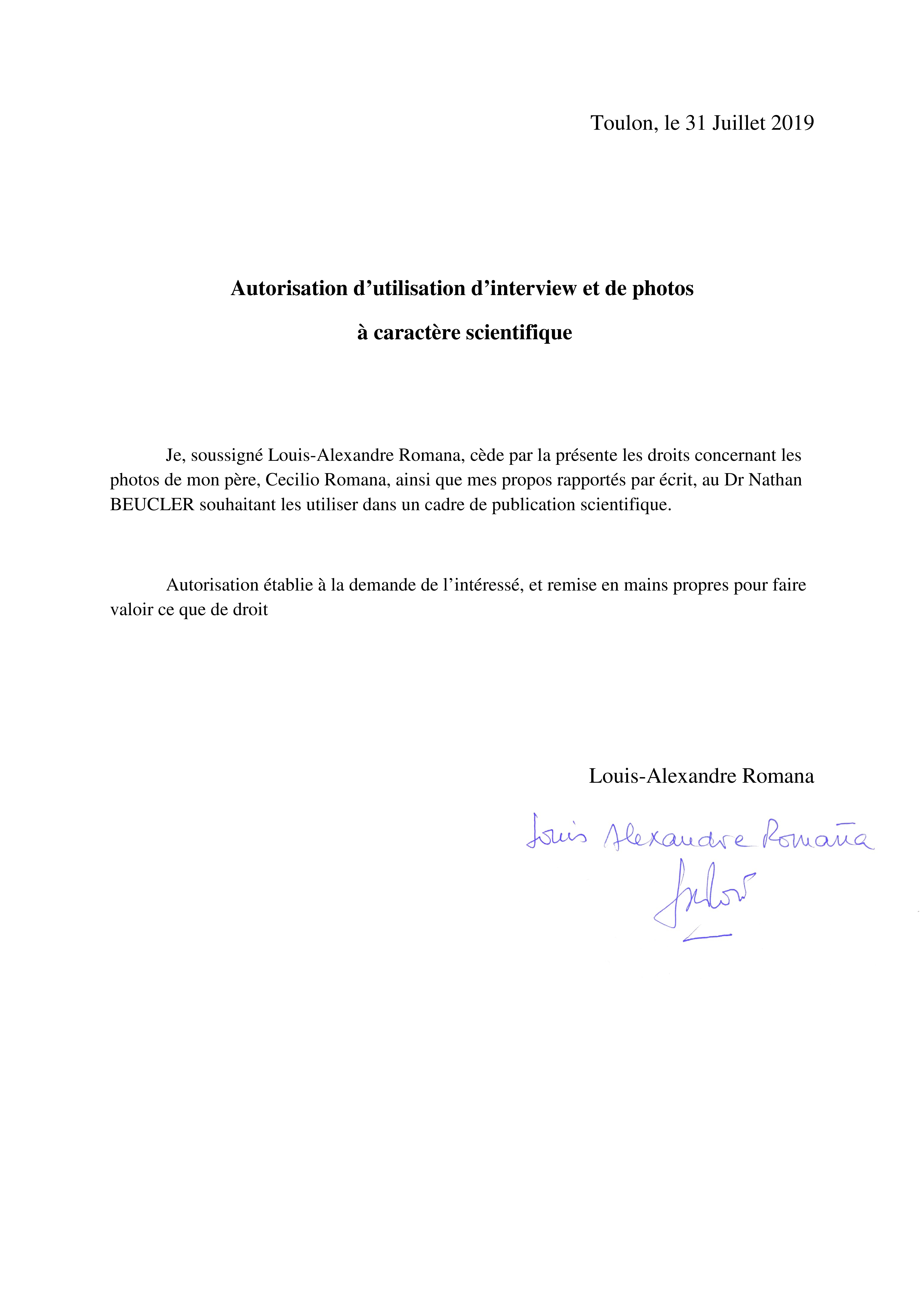

Supplement: S1 Authorization — (JPG) [file pntd.0008836.s002.jpg]
